# Functionalities and Issues in the Implementation of Personal Health Records: Systematic Review

**DOI:** 10.2196/26236

**Published:** 2021-07-21

**Authors:** Nabila Clydea Harahap, Putu Wuri Handayani, Achmad Nizar Hidayanto

**Affiliations:** 1 Faculty of Computer Science University of Indonesia Depok Indonesia

**Keywords:** personal health record, systematic review, functionalities, issues

## Abstract

**Background:**

Functionalities of personal health record (PHR) are evolving, and continued discussions about PHR functionalities need to be performed to keep it up-to-date. Technological issues such as nonfunctional requirements should also be discussed in the implementation of PHR.

**Objective:**

This study systematically reviewed the main functionalities and issues in implementing the PHR.

**Methods:**

This systematic review was conducted using Preferred Reporting Items for Systematic Reviews and Meta-analyses (PRISMA) guidelines. The search is performed using the online databases Scopus, ScienceDirect, IEEE, MEDLINE, CINAHL, and PubMed for English journal articles and conference proceedings published between 2015 and 2020.

**Results:**

A total of 105 articles were selected in the review. Seven function categories were identified in this review, which is grouped into basic and advanced functions. Health records and administrative records were grouped into basic functions. Medication management, communication, appointment management, education, and self-health monitoring were grouped into advanced functions. The issues found in this study include interoperability, security and privacy, usability, data quality, and personalization.

**Conclusions:**

In addition to PHR basic and advanced functions, other supporting functionalities may also need to be developed based on the issues identified in this study. This paper provides an integrated PHR architectural model that describes the functional requirements and data sources of PHRs.

## Introduction

In health emergencies such as epidemics, natural disasters, or artificial disasters, access to reliable health information becomes crucial for the community [1,2]. As of 2020, the COVID-19 pandemic throughout the world has led to an increasing need for electronic health records (EHRs) to provide reliable health information [3,4]. According to the World Health Organization, the EHR that collects data from various health service providers will provide better patient care during a pandemic, such as preventing and detecting an outbreak [5]. The EHR’s function will be more optimal if patients can share their health data with health care providers [6]. Personal health records (PHRs) can help patients share their data with health care providers and provide useful information during health emergencies [2].

The EHR aims to collect health data managed by health care providers, while the PHR aims to collect health data entered by individuals [1]. The PHR was developed with a patient-centered approach in the capture and storage of information [7]. In its simplest form, a PHR is a stand-alone application that is not connected to other systems. Users can access their PHR using commercially available applications to record and analyze daily activities and habits to maintain a healthy lifestyle. In a more complex form, the PHR’s health information is connected to the EHR of the health care provider (tethered PHR) or to various health service data sources (integrated or interconnected PHR). A PHR integrated with an EHR, either through tethering or interconnectivity, provides far more significant benefits than a stand-alone PHR [1].

One of the important PHR research areas is PHR functionality [8]. Previous studies have provided data types and functionalities of PHRs [9] and a guide to evaluate PHR functionalities [10]. Some studies reviewed PHRs used for chronic diseases, which include discussions about their functionalities [11,12]. However, these studies focused only on PHRs in the United States and developed countries [9,11,12]. Moreover, previous studies have also discussed technological issues in implementing or using PHRs, such as data quality [13], personalization [14], privacy [13,15], and usability [14]. These studies still have no clear explanations about how these issues can be included as requirements in implementing PHRs.

Functions or features of PHRs are evolving [9,12], so continued discussions about PHR functionalities need to be held to keep the research up-to-date. In addition, technological issues as nonfunctional requirements [16] in the design and development of a system must be discussed. Technological issues can be defined as constraints and qualities related to the technology used to perform the function [17]. Thus, this paper aims to review the PHR studies focusing on the functionalities and technological issues in building the PHR system. This paper addresses the following research question: What are the main functionalities and issues in the implementation of PHRs? This study can provide PHR design or implementation recommendations to health care management, application developers, policymakers, or other related stakeholders.

## Methods

This systematic review was conducted using the Preferred Reporting Items for Systematic Reviews and Meta-analyses (PRISMA) guidelines [18]. PRISMA is suitable for studies related to health care interventions, and it focuses on ways in which authors can ensure the transparent and complete reporting of systematic reviews [19]. The PRISMA checklist for this study is provided in Multimedia Appendix 1.

### Search Strategy

The search is conducted using the online databases Scopus, ScienceDirect, IEEE Xplore, MEDLINE, CINAHL, and PubMed. Terms or keywords used to search the articles: (“phr” OR “personal health record” OR “personal medical record” OR “personal health information” OR “personally controlled electronic health record” OR “pcehr” OR “patient portal”) AND (“functionality” OR “features” OR “issues” OR “implementation”). The search was conducted for journal articles and conference proceedings published between January 2015 and December 2020 to ensure that the data were current because the functions of PHRs are evolving.

### Eligibility Criteria

The authors defined inclusion criteria as the review guidelines for study selection. The articles included for this study must have full text available and written in English, be original research articles, focus on discussing the electronic PHR platform, and discuss functionalities and/or issues in the implementation of PHRs.

In this study, the PHRs discussed are all PHR types (stand-alone, tethered, and integrated) that provide access to health information or records to patients electronically. Therefore, papers with related terms such as patient health records or patient portals are also included in this review. The authors also reviewed PHRs at the design stage to include conceptual papers in this review.

### Study Selection

The study selection consists of the following phases:

1. Keyword or search string was searched in each online database previously mentioned. Duplicated records were checked and removed.
2. The title and abstract of identified articles were selected based on the eligibility criteria. Articles that did not meet inclusion criteria were eliminated.
3. Articles that were not eliminated in the previous stage were read in full text to determine whether they should be included in the review based on the eligibility criteria. Reference lists of the included studies were also checked to identify additional relevant articles.

The first author screened the titles and abstracts based on the eligibility criteria. The same author reviewed full-text versions of the articles that were not excluded from the previous screening. The first author extracted data from selected studies and the second author reviewed the extracted data. Disagreements between the two authors were resolved through discussion. If an agreement could not be made, the third author would determine the decision. We were unable to consistently evaluate the risk of bias due to the variety of methodologies within the studies.

### Data Items and Synthesis

Data collection was performed manually using a data extraction form. Information extracted from each article consists of characteristics of selected articles, such as study location, PHR purpose, and methodology, and functionalities of PHRs and issues in PHR implementation

Authors categorized functionalities of PHRs based on their purpose as defined in Bouayad et al [9], Price et al [12], and Genitsaridi et al [10]. For each function category, the authors explained subfunctions or data elements that were implemented or recommended from the selected articles. Moreover, each function category was grouped based on basic and advanced functions defined by Detmer et al [20]. Basic functionalities help people collect, organize, and store health information, while advanced functionalities enable patients to play a more active role in their health [20]. The authors explained PHR implementation issues that are mentioned explicitly or implicitly from the selected articles.

## Results

### Study Selection

**Figure 1:**
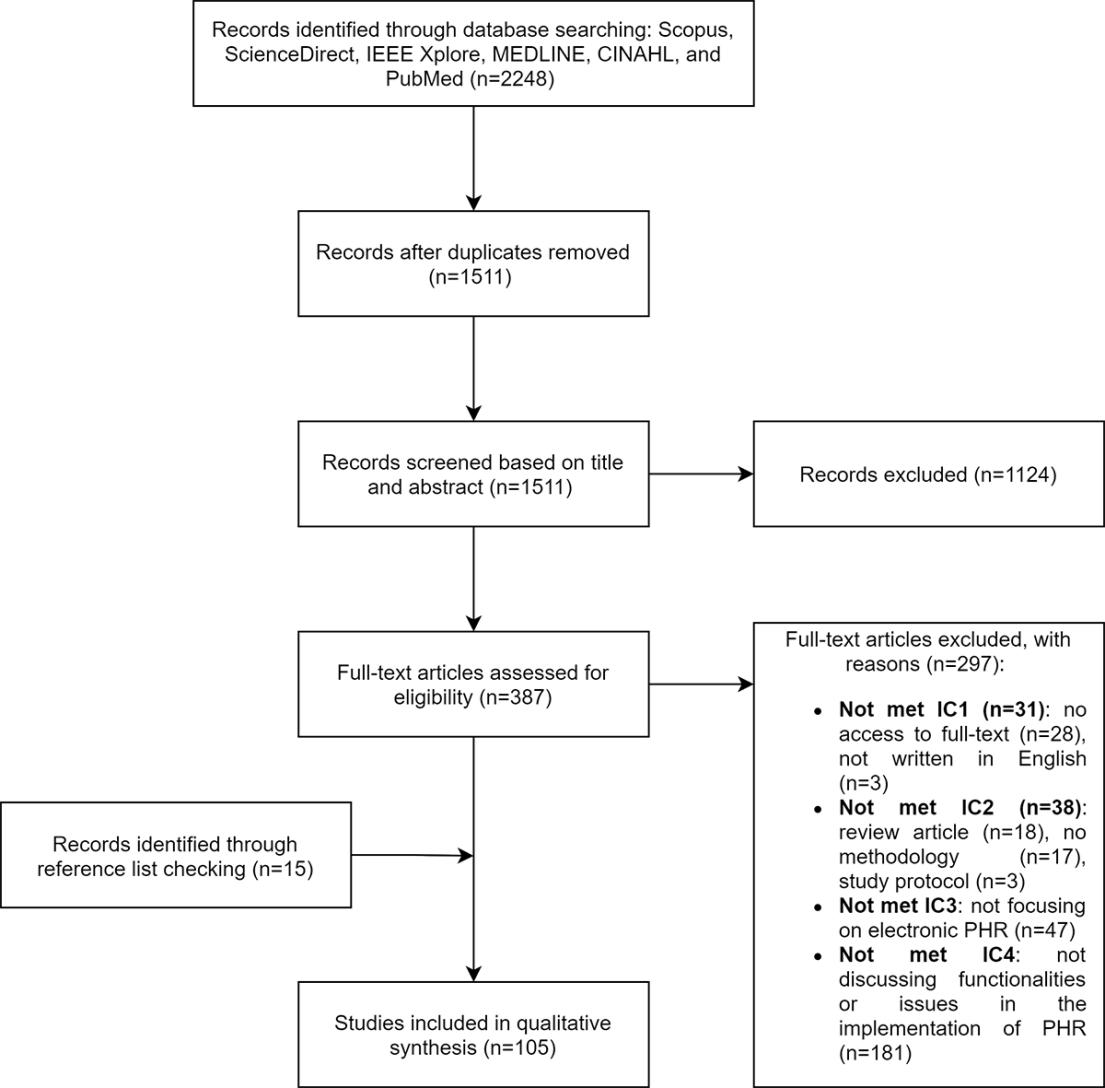
Flow diagram for search results. IEEE: Institute of Electrical and Electronics Engineers; MEDLINE: Medical Literature Analysis and Retrieval System Online; CINAHL: Cumulative Index to Nursing and Allied Health Literature; PHR: personal health record.

The database search results identified 2248 studies from 2015 to 2020. Next, duplicate records were removed, resulting in a total of 1511 studies; 124 studies were excluded after the title and abstract screening (articles that mentioned literature review and articles not related to PHRs, patient portals, or access to health records to patients were excluded at this stage). A total of 387 articles were assessed in full text, of which 297 were excluded because they did not meet the selection criteria. However, 15 additional studies were identified from reference lists checking, for a total of 105 studies included in this review (Figure 1).

### Study Characteristics

**Figure 2:**
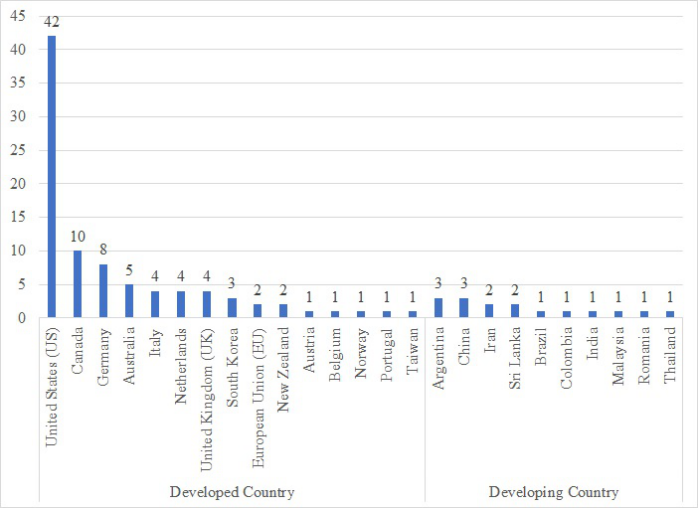
Countries involved in personal health record study.

The chosen articles showed that PHR research has mainly been done in developed countries such as the United States, Canada, and European countries compared to developing countries. This country classification was based on the United Nations World Economic Situation and Prospects 2020 [21]. Countries involved in selected studies consist of developed countries such as the United States (42 studies), Canada (10 studies), Germany (8 studies), Australia (5 studies), Italy (4 studies), Netherlands (4 studies), United Kingdom (4 studies), South Korea (3 studies), European Union (2 studies), New Zealand (2 studies), Austria (1 study), Belgium (1 study), Norway (1 study), Portugal (1 study), and Taiwan (1 study) and developing countries such as Argentina (3 studies), China (3 studies), Iran (2 studies), Sri Lanka (2 studies), Brazil (1 study), Colombia (1 study), India (1 study), Malaysia (1 study), Romania (1 study), and Thailand (1 study; Figure 2).

The purposes of PHRs (Table 1) in selected articles include general, not specific to the disease, health status, or population (48 studies); chronic diseases such as cancer, cardiovascular disease, and diabetes (31 studies); hospital patients such as inpatients and outpatients (10 studies), older adults (5 studies), women and child health (4 studies), mental health (4 studies), and other specific populations such as employees and foster youth (3 studies).

The study methods (Table 2) used in selected studies include qualitative (41 studies), quantitative (33 studies), conceptual paper (16 studies), and mixed method (15 studies). A summary table of the characteristics of the included studies is provided in Multimedia Appendix 2.

**Table 1:** Purposes of PHRs^a^.

Category	Description	Number of studies
General	PHR designated not specific to any diseases, health status, or population.	48
Chronic disease	PHR for chronic diseases such as cancer, diabetes, or cardiovascular disease.	31
Hospital patients	PHR for patients who have visited the hospital, such as inpatients and outpatients.	10
Older adults	PHR for patients with the age of more than 50 years.	5
Women and child health	PHR for women, pregnancy, and pediatric health.	4
Mental health	PHR for mental health diseases, such as bipolar disorder.	4
Other populations	PHR for other specific populations, such as employees and foster youth.	3
Total	—^b^	105

^a^PHRs:patient health records.

^b^Not applicable.

**Table 2:** Methods used in the studies.

Method	Type of study	Number of studies
Qualitative	Interview and focus group discussion	41
Quantitative	Questionnaire, cohort study, and randomized clinical trial	33
Conceptual paper	—^a^	16
Mixed method	—	15
Total	—	105

^a^Not applicable.

### Main Functionalities of PHR

Basic functions identified in this study consist of the health record and administrative record. Advanced functions consist of medication management, communication, appointment management, education, and self-health monitoring (Table 3). A summary table of the data elements and subfunctions is provided in Multimedia Appendix 3.

**Table 3:** Identified Functionalities in PHR^a^.

Function		Description	References
**Basic function**			
	Health record	Allows patients to view or access clinical documents from health providers’ EHR^b^.	[22-71]
	Administrative record	Allows patients to manage personal information and view information related to health providers and insurance.	[22,25,26,30,31,34,39,42,44-46,55,59,66,68,70,72-78]
**Advanced function**			
	Medications management	Allows patients to manage information related to medications and prescriptions.	[24-36,38-40,42,45,46,48,51-57,59-61,63,68-71,73,74,77,79-85]
	Communication	Allows patients to interact and communicate with health care providers and others, such as support groups and families.	[22-24,27-32,36,38,40,42,43,46,48,49,51-53,55-57, 59,61,62,67,69,71,74,75,80,82-84,86-94]
	Appointment management	Allows patients to manage appointments with health care providers.	[22,23,25-31,33,34,36,40,42,47-53,55-57, 59-61,63,64,67,71,73,77,78,81,85-87,92,94]
	Education	Allows patients to access health-related education resources.	[22,30,31,40,45,46,55,57,59,61,70,71,76,77,90,95-99]
	Self-health monitoring	Allows patients to manage their self-health data through clinical measures.	[23,26,30,33,39,44,58,66,67,70,72,81,85,86,90,93,95,96,98,100-105]

^a^PHR: patient health record.

^b^EHR: electronic health record.

### Basic Functions

#### Health Record

**Figure 3:**
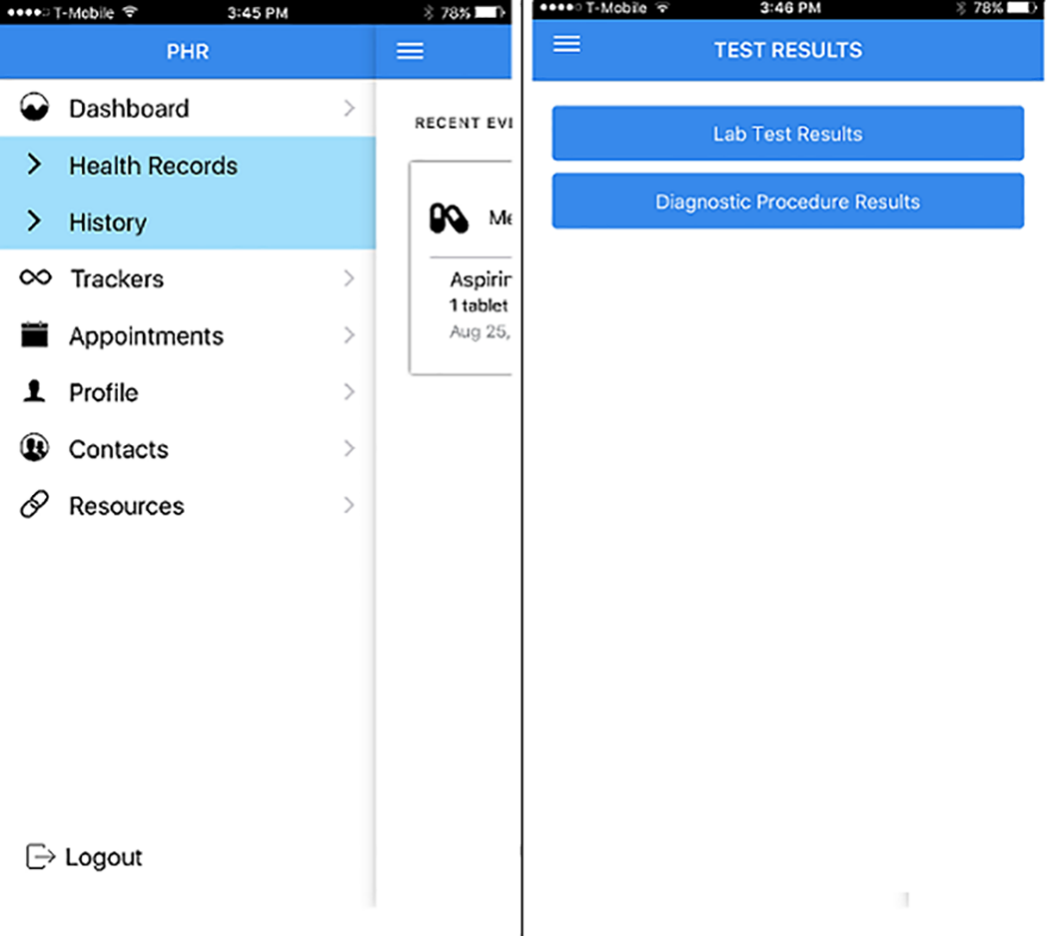
Test results menu in the health records module of PittPHR [33].

The health record function provides patients options to view clinical documents that can be retrieved from health providers’ EHR [22,23,34]. This information can include problem lists [22,24-29,45,56,67-71], allergies [22,25-28,30-33,35-39, 56,67-70], immunization [22,26-28,30,32-35, 40-42,56,68-71], laboratory and test results [22,24,26-30,33,36,37, 40,42-61,67-71], diagnostic information [32,37,44,45,62], discharge information [31,34,63], and clinical notes [24,30,33,42,50,61,64,67,69]. Figure 3 shows an example of test results in PittPHR [33].

This function can also include information about medical history [22,24,30,33,35,37,39,45,51,57,64,65,67,68], family history [28,30,32,33,35,37,66], genetic history [45], surgical history [26,28,33,35,45,66,68], social history [32,33,35,37,45,68]. Some studies suggested that this function also supports patients’ ability to print the record [24,36] and add comments or notes in health records [28,61].

#### Administrative Record

This function enables the patient to manage information about demographics or personal information [25,26,31,44,45,68,72] such as name [25,39,44,72,73], gender [25,26,44,72], birthdate [25,26,44,72,73], blood type [39,44], contact information [25,31,68,72], and parents’ names [25,45]. Patients can also change their information, such as change password, address, and email address [22]. Patients can enter this information [22,31,68,72] or retrieve it from a central patient registry, such as in Lifelong PHR [34].

**Figure 4:**
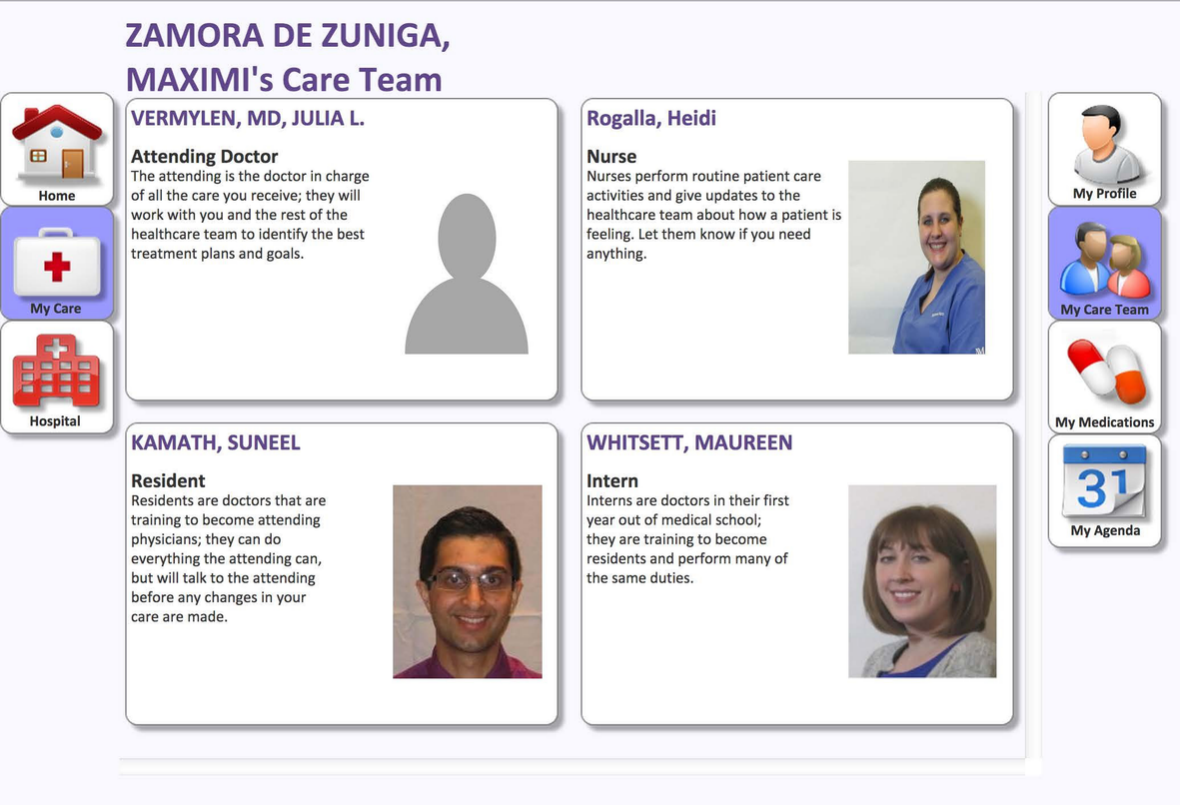
Health care team information menu [74].

Patients can also view health professionals’ information, such as name of health worker [34,39,55,74], role [74,75], educational background [75], contact information [42,45], specialty [76], location [76], and pictures [46,55,59,74,77,78]. Patients can also view hospital information, such as location, contact info, address, navigation [70]. Patients can also view and pay bill [42,55,77] and get insurance-related information [22,30,34,45,66]. This data can be retrieved from the regional health care information system [34]. Figure 4 shows an example of the health care team information in the PHR app [74].

### Advanced Functions

#### Medication Management

Health care providers publish prescriptions to the patient’s PHR, while pharmacists dispense the prescribed medication [34]. This function provides information about the list of medications that patients are currently taking [25,28,29,35,38,45,46, 52,56,59,60,68,69], medication name and dosage [32,35,40,60,74,77,79,80], and list of past medications [28,29,36,42,45,46,60,69,70,73,81]. PHRs should also add information about the purpose or class of medications to give patients an understanding of the medication type [74] and allow pharmacists to explore the data according to their common questions [80].

**Figure 5:**
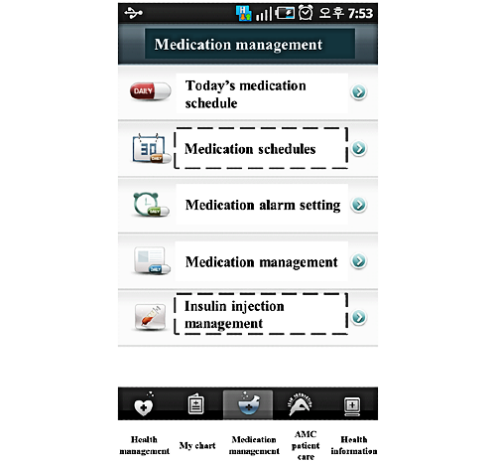
Medication management in My Chart in My Hand [85].

This function also allows patients to view list of prescribed medications [26,30,39,54,79,82,83], prescribing physician [79], refill prescription [24,27,29-31,33,34,36,39,48,53,55, 57,60,61,84], order medications [29,39,71], deliver purchased medication [79], as well as track the delivery of medication [36,39,42]. Some PHRs also provide medication schedulers and reminders of when to take medicines [28,70,83,85], drug or medicine reconciliation [42,51,63,83], and warning alerts of potential adverse interactions based on the medication and allergy list [38,68,73]. Figure 5 shows an example of medication management in medication management in My Chart in My Hand [85].

#### Communication

**Figure 6:**
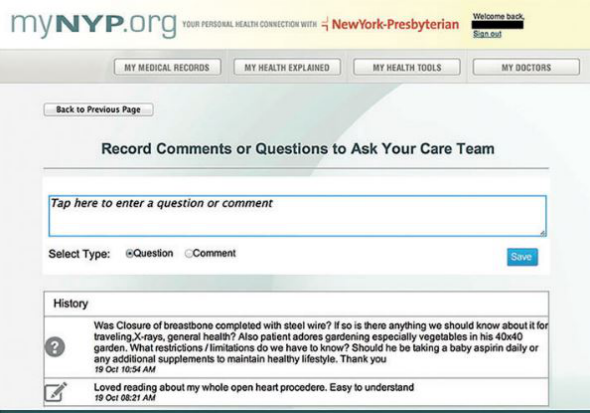
Comments or questions page in myNYP.org [80].

The patient can send messages to the health care provider to inform them of health condition [23], share doubts and worries [86], receive medical advice [56,86], or send nonurgent messages [40,46,71]. The communication can be in the form of messaging [23,27,29-32,36,38,40,42,43,46,48,49,51-53,55-57, 59,61,67,69,71,74,75,82-84,86-92] or text where patients can write questions (Figure 6) [74,80]. Some PHRs also enable patients to contact others in a similar situation [28,29,83,88], support groups [62,87], family [75,89], or customer support and billing departments [22]. Some studies also suggested this function have the ability to maintain a record of past conversations [36] and provide email or text notification when a health care provider leaves a message on the PHR [24,93,94]. Moreover, some studies suggested tracking the status of a question [80], message multiple providers at the same time [24], and import selected emails and interactions on the social network to PHRs [86].

#### Appointment Management

**Figure 7:**
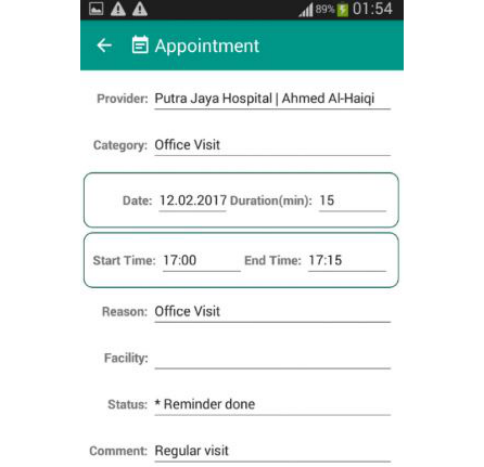
Appointment scheduling in mPHR [25].

Some PHRs may allow a patient to request or schedule appointments (Figure 7) [22,23,25-28,30,33,34,40, 42,48-50,52,55,57,60,61,67,71,85,87,92], while others only allow patients to view their past and upcoming appointments [29,31,36,51,53,56,59,63,64,73,77]. The types of appointments can include patient-doctor visit consultation services and other health services such as specialist encounters, sample takings, hospital admissions, result withdrawal [86], therapies, and online consultation [23]. Moreover, some studies suggested that PHRs include reminders or notifications for upcoming appointments [33,42,47,48,60,61,81,94]. This reminder can be in the form of email notifications about the date and time of the appointment [42,81]. PHRs can also add a calendar to keep track of future appointments [34,78].

#### Education

**Figure 8:**
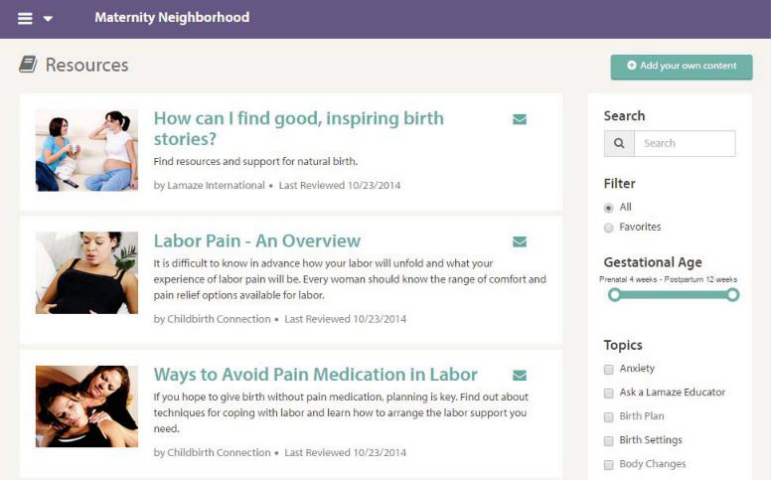
Education resources in Maternity Information Access Point [97].

The education function can include resources from trusted websites [45,90], health information libraries [22,30], video resources [46,59,95], or government supported information [95]. The information can consist of lifestyle management [45,57,71], first-aid information [40,70], discharge instructions [31], surgical procedure [77], physical activities guidance [96], or health-specific education such as pregnancy [97,98], mental health [45,61], or chronic diseases–related education [90,95]. Figure 8 shows an example of the education page in the Maternity Information Access Point [97]. Health providers are responsible for providing clinical topics and resources for credible information [55,76,99]. Moreover, PHRs should also have the ability to search for information using an intelligent search engine [99].

#### Self-Health Monitoring

Patients can manage their own health related to nutrition and diet information such as weight [30,33,39,58,66,67,72,85,86,90,98,100-102], height [39,58,66], physical activity or exercise [30,33,58,66,70,96,98,100,101], and food and meals [33,66,98]. Patients can also manage their vital sign data such as temperature [26,44], blood pressure [30,33,44,58,66,67,70,72,85,90,98,101,103], blood glucose [30,58,66,70,72,85,86,98,103], and heart rate [90]. Patients can also monitor other self-health data such as sleep [33,66,95,100,101], period [33,100], moods [98,100,101], and stress [66,70,100].

These clinical measures enable calculation such as BMI [39,66,70,85,100], body fat percentage [70], waist-to-height ratio [70], calorie [70], cholesterol level [66,86], and glycemia [86]. This information can also calculate disease risks such as cardiovascular disease risk and metabolic syndrome risk [85]. The data in this function can be retrieved from home monitoring devices [23,39,58,85,96,104,105] such as Bluetooth-enabled health monitors [104], accelerometers [105], blood pressure monitors [58], blood glucose meters [58,81,85,93], and pedometer [72] and fitness tracker apps [39].

**Figure 9:**
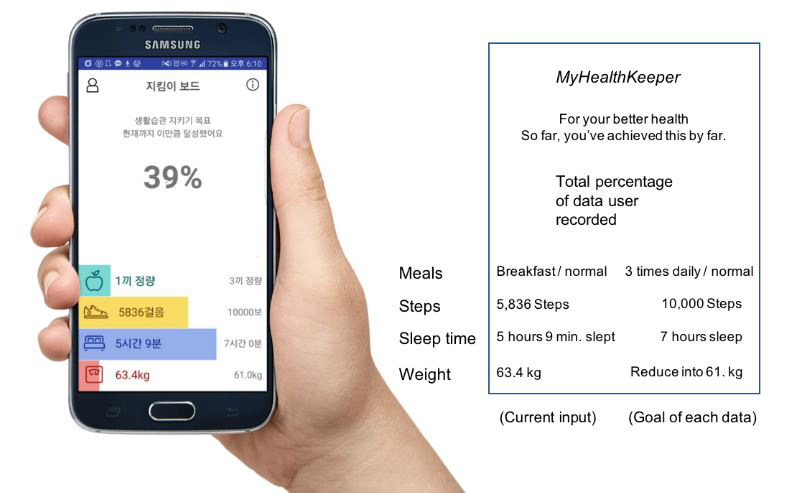
MyHealthKeeper interface for patient’s lifestyle data [101].

The monitoring of health data can be shown as a dashboard that visualizes data in graphs, charts, or diagrams [30,33,70,72,81,86,101,102,105]. Key performance indicators can be different for each patient, depending on their conditions. For example, in the MyHealthKeeper app, the clinician provided individual diet and physical activity targets for each patient during an outpatient visit (Figure 9) [101]. This function can be integrated into a clinician’s EHR, and clinicians could review these data and provide feedback about the health-related lifestyle management of their patients [101].

### Issues in Implementation of the PHR

Some issues must be considered in implementing PHRs because these issues can define additional functionalities that can support the main functionalities in PHRs. The issues identified included interoperability, security and privacy, usability, data quality, and personalization (Table 4).

**Table 4:** Issues in implementation of the PHR^a^.

Issues	Description	References
Interoperability	Ability of PHR to share or exchange data with other systems	[22,25,29,33,35,37-39,51,53,61-63, 68,72,76,86,88-90,96,100,102,104,106-112]
Security and privacy	Safeguarding of data and personal information in PHR	[25,26,32-34,42,45,47,54,55,57,60-65,69,70,72-74,76,77,83, 92,94,97,103,108,110,112-122]
Usability	Whether users can use PHR effectively and efficiently	[24,25,33-37,41,45,46,48,51,54,59,61,62,65,71,74, 76,81,83,85,87,90,92-94,96,100,105,108,110,117-126]
Data quality	Ensures consistency, completeness, accuracy, and timeliness of the PHR information	[24,30,31,33-35,64,68,81,90,91,107,108,110-113,115]
Personalization	Ability of PHR to be tailored and adapted to patient needs and preferences	[22,33,52,57,59,78,87,88,95,99,102,112,117,118,123,125]

^a^PHR: personal health record.

#### Interoperability

An important issue raised in several studies is PHR compatibility with other systems [51,88,90,100,106]. Health service providers such as clinicians should input data from other systems into the PHR or vice versa, which was considered too time-consuming and unfeasible for daily practice [100]. This problem can also be caused by health organizations adapting their formats to use health records and not allowing health information sharing in their PHR to other applications or organizations [63,107]. As a result, a patient may have health records scattered in several applications [107]. To provide more benefits and ensure its successful implementation, PHRs should realize interoperability among various data and systems [106,108]. PHRs should have the ability to share information with others [88], such as health professionals [37,61,89].

In the tethered or integrated PHR, patients may connect their PHR to the health care provider system [25,62,72,76,86,102,104]. With this integration, health information is automatically transferred to the PHR [33,39,62,109,110]. This can reduce data entry load [33,96], improve data accuracy [62,96], prevent medical errors [38], reduce the health information recall [35], and contribute to users’ better perceptions about the system’s usefulness [111]. It is also suggested that PHRs be integrated into various health providers and not limited to one health provider [53,102]. Patients may also have the ability to share information with trusted institutions and insurance bodies to speed up reimbursement procedures [86] and access other family members’ records [22,102].

It is necessary to create legislation to realize PHR interoperability [106]. Health providers need to provide standard definitions for data exchange and cooperate with other providers [63]. There are international standards or frameworks for interoperability, such as OpenEHR, Health Level 7 (HL7) Fast Healthcare Interoperability Resources (FHIR), and Integrating the Healthcare Enterprise and Continua Health Alliance specifications [29,68,72,107,112]. OpenEHR describes the management and exchange of data in EHRs for developing PHRs using specific language [68]. OpenEHR integrated with other standards in particular health data types, such as laboratory results [107]. Similarly, HL7 FHIR enables the management of a single data entity, group of entities, or a record using well-known standard languages [68]. FHIR application program interface allows any arbitrary system connected with another medical system already equipped with the FHIR application program interface [72]. FHIR allows the patient portal to be interconnected but independent [29]. Moreover, Integrating the Healthcare Enterprise specifies architectural approaches using international standards for the health data exchange and can fit the mobile platform’s resources. At the same time, Continua enables communication from personal health devices to EHRs and PHRs [112].

#### Security and Privacy

PHRs contain personal and sensitive data [47,77,108,112-114]. Some people have concerns about storing these data online [54,103,108,113,115] and consent to use the system [116]. They may have concerns about identity theft and unauthorized access in PHRs [54,55,57,61,69]. Confidentiality and privacy of information in PHRs should be ensured through secured access to PHRs [110].

To ensure the security of information, PHRs should use a single sign-on mechanism [70], user authentication [26,33,64,72,73,112,117], authorization [42,112], identity verification [34,63], encryption [25,33,112,118] or pseudonymization [114], backup mechanism [25,33,72], and firewalls [72]. PHRs can also implement an access log so that users can see who viewed and downloaded information [76]. The use of complicated or complex passwords can improve the security of s [47,119]. However, some studies show that users have difficulty remembering their passwords [47,74,77,92,94,97,103,120]. Thus, PHRs should also add other methods such as fingerprint authentication [97], biometric identification [33,94], citizen digital certificate [121], and allow users to change their passwords [62,72].

To address privacy concerns related to data sharing, PHRs should have the ability for patients to choose what information to share and who can see that information [34,42,45,60,61,65,76,83,121,122] and provide a privacy policy in the system [32]. The consent model should also be considered in implementing PHRs [116]. Moreover, PHR systems need to follow specific legal requirements related to security and privacy defined on regional, national, or international levels [112]. For example, the Health Insurance Portability and Accountability Act ensures secure data exchange with entire clinics [76].

#### Usability

Some usability problems identified in selected studies include font or text size that are difficult to use [71,94], confusing format [81], unclear visualization of data [90], problem with navigation [51,59], and complicated data entry [85,118]. Complicated data entry may cause users to not enter data correctly into their PHR [118]. The reduction and simplification of PHR system data entry should be considered in PHR design [35,93,118]. Users prefer easy to use, simple, and user-friendly interface [24,41,45,54,61,62,65,92,94,110,118,120-124]. Users are also interested in attractive and interactive systems [25,33,108,110,120,124] such as the use of contrasting colors for scroll bars and menu items [59]. Moreover, it is also important to maintain consistency and standardization of interfaces [35,74,117,118]. A mobile app version of the PHR was also suggested because it was perceived as more user-friendly and easy to use [25,34,61,65,81,93,100].

A PHR may add a section to guide patients about the features in the PHR [46,54,59,61,65,83,90,96,118] and quick access to the essential functionalities [37,48,108]. The use of user-interface elements like buttons and a dropdown menu can enhance the user-friendliness and simplicity of the PHR interface [25,100]. However, icons should be avoided when designing for older adults since they may not recognize them [96]. PHRs should be easy to understand and navigate for all user groups [110], including those with basic computer knowledge and those who are not computer literate [94,118]. PHR usability should be determined using health literacy assessments and there should be different PHR versions for specific groups of users [36].

Developers should involve users in designing, updating, or improving PHR systems [48,71,119]. Using a user-centered design approach can facilitate users’ involvement in PHR design [76,87]. The user-centered design process increased the development process’s complexity, but the product quality was higher, especially satisfaction and user acceptance [105]. However, user-centered design may not apply to all PHR types, especially PHRs targeting the general population, which necessitates identifying specific user groups and specific use contexts [125]. Adopting a usability design framework that includes usability and user testing may help address PHR usability issues [126]. Standardization used for PHR design is International Standards Organization (ISO 9241-210), which focuses on the requirements and user needs [105], and ISO 9241-11 for software systems components that define usability [96].

#### Data Quality

Health care providers may doubt patient-entered data in a PHR [30,113]. Not all patients have enough knowledge to generate health data in a PHR [107,112]. Data uploaded by the patient may be inconsistent [91], incomplete [81,90,110], inaccurate [30,81,90,110,115], or not up to date [90]. PHRs require patient commitment to keep the system up-to-date and relevant over time [111]. This issue needs particular attention, especially when PHR data are transferred to EHRs and used in professional medical decision-making and treatment processes [112].

To ensure data quality of patient-generated data in PHRs, health care professionals need to take time to supervise the quality of information generated by patients in PHRs [110,112]. PHRs should differentiate patient-generated data from the health care provider’s data [68]. Moreover, PHR design needs to define what information is required because an incomplete record is preferable to an inaccurate one from a provider’s perspective [35]. Standardization of patient-entered information is essential to ensure data quality [33]. Input control should be comfortable and descriptive words should appear to help patients enter PHR data [108].

In tethered PHRs, which are tied to EHRs in health organizations, health information on the PHRs are created automatically from the original patient clinical reports to make this information more reliable [34]. However, this can be a problem if the EHR’s information is incomplete [30,64] or if the information is not generated automatically. This can also be caused by health care providers not updating the PHR information consistently [24,31].

#### Personalization

Some users may have more health issues than others, such as older patients having more health issues, appointments, and information to manage [22]. People want the PHR to be tailored to their needs and capable of changing based on their health and well-being needs [59,78,88,102,123]. This person-specific health and well-being information can make the PHR system more appealing [118]. The PHR system needs to be adaptable and extensible to ensure successful operation [112]. It is also suggested that PHRs support customizability based on computer literacy [87].

PHR systems should provide medical information that can be dynamically adapted to patient preferences for simpler or more complex information [99] [117]. For example, in PittPHR, users can customize the trackers according to their own needs by hiding or unhiding available trackers in a given list and add or delete links in the resources module according to their own needs [33]. PHRs could also provide tailored health education materials based on patient health problems [52,57,95,117]. Despite the need for personalization, designers or developers need to define the extent to which PHRs can be personalized but still maintain standardization, uniformity, and simplicity [125].

## Discussion

### Principal Findings

Seven function categories of PHRs are identified as the main functionalities of PHRs, which are grouped into basic and advanced functions. Basic functions (health records and administrative records) provide essential information for patients in their health care. Health records could provide a complete summary of patient health status and condition. Information on this function could reduce health workers’ time gathering patient history and reduce redundant transactions and tests [20]. Information on administrative records such as personal information serves as a patient identifier on a PHR.

Advanced functions (medication management, communication, appointment management, education, and self-health monitoring) could support patient involvement in their health care. Involving patients in controlling their health information improves the chance that health providers would have a comprehensive view of patient health conditions [20]. Medication management functions such as medication scheduler and reminders could help patients take medicine on time. Moreover, the medication reconciliation option could avoid medication errors [127]. Communication functions such as messaging could free physicians from the limitations of phone and face-to-face communication [1]. Appointment management reduces the chance of a patient missing an appointment. Education could support health knowledge promotion [20], which may improve the patient’s health literacy. The information recorded from the self-health monitoring function may help health providers with disease diagnosis and treatment [10]. This function could help patients track their progress to reach specific health goals [9] and monitor the impact of their behavioral changes [12].

In addition to these functionalities, other supporting functionalities may also need to be developed based on the issues identified in this study. To improve security and privacy, PHRs should implement access control, which includes authentication and authorization. PHRs can also provide a backup option to avoid data loss and audit logs to review who accessed the record and what data have been accessed. To improve usability, PHRs can provide quick access to the important information or functions that users frequently use and add a menu for help or a user guide about using features in the PHR. Customization options to show or hide specific health data according to patient health needs are also recommended to increase personalization.

**Figure 10:**
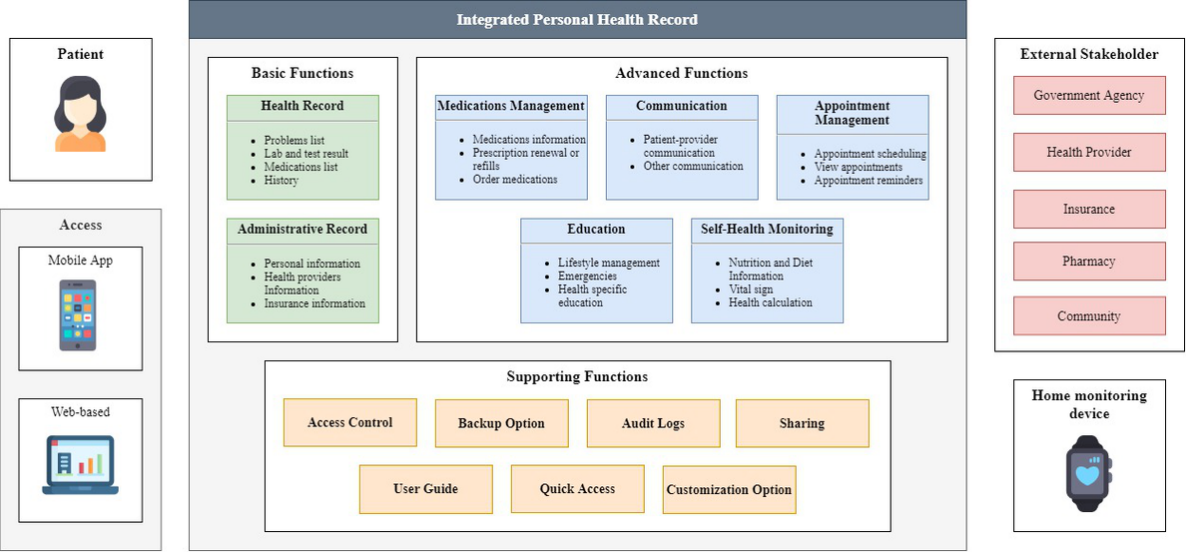
Integrated personal health record architecture.

Interoperability represents a key component of PHR architecture [8]. When PHRs are integrated with health providers, they provide more significant benefits and valuable content for users [1,20]. Interoperability can also reduce data entry load because health information is automatically transferred to the PHR. This can increase the usability and the quality of data on the PHR. PHRs need to provide standard definitions for data exchange and implement sharing functions to connect PHRs with other stakeholders, such as health providers, insurance, government agency, pharmacy, community or support groups, and other systems such as home monitoring devices. Figure 10 describes the integrated PHR architecture based on the result of this review.

The main functionalities described in this review, such as health records, administrative records, medication management, communication, appointment management, education, and self-health monitoring, have also been described in previous reviews [9-12]. Most of these reviews [9,11,12] focus more on discussing the functionality of PHRs related to improving health service delivery. Only Genitsaridi et al [10] discussed supporting functionalities such as access control to be included in requirements on the PHR. Previous studies also have discussed technological issues [13-15]. Our research augments that of previous studies by translating these issues as supporting functionalities in PHR systems.

The functionalities in PHRs can help health care providers and patients obtain useful health information during public health emergencies such as natural disasters and pandemics. For example, in the COVID-19 pandemic, hospital services experienced a crisis [128]. Observations of health outside of standard hospital settings can be difficult [129]. Functions such as communication can help patients and health care providers consult without making eye contact. Furthermore, in the education function, PHR providers can provide information about updated COVID-19 and health care information. The health record function helps patients obtain and store test results. The self-health monitoring function increases the patient’s ability to control and manage health conditions. Functions such as measuring body temperature can be used to detect early signs of infection [129]. Integrating PHRs into a broader telehealth infrastructure could improve emergency health care delivery by reducing patient spikes in health care facilities [2].

### Comparison With Prior Work

The Health Level 7 Personal Health Record System Functional Model (HL7 PHR-S FM) defines a standardized model of the functions present in PHR systems [130,131]. The model consists of 3 sections: personal health (PH), supportive (S), and information infrastructure (IN). Personal health functions enable an individual to manage information about their health care. Supportive roles assist with the administrative and financial requirements within health care delivery. Information infrastructure functions support personal health and supportive functions.

Health records, medication management, communication, education, and self-health monitoring can be categorized into personal health sections. Administrative records such as managing patient profiles can be categorized into a personal health section, while information about health professionals, hospitals, and insurance can be categorized as a supportive section. Supporting functions defined based on PHR implementation issues, namely sharing, access control, audit logs, backup options, and customization, can be categorized in the information infrastructure section. This section ensures the privacy and security of PHRs, promotes interoperability between PHRs and other systems, and enables PHR function to be accessible and easy to use [130,131]. Table 5 summarized comparisons between functions identified in this review study and functions defined in the HL7 PHR-S FM.

**Table 5:** Comparisons between functions.

Functions identified and ID				Functions defined in the HL7 PHR-S FM^a^		
				Function name		Description
**Basic functions**						
	**Health record**					
		PH.2.5	Manage historical and current state data		Provide a summary of the patient’s current medical state and history	
	**Administrative record**					
		PH.1.2	Manage PHR^b^ account holder demographics		Capture the patient’s demographic information	
		S.1.3	Manage health care provider information		Import or retrieval of data necessary to identify a health care provider	
		S.1.5	Manage health care facility information		Import or retrieve of data necessary to identify a health care facility	
		S.2.1	Capture and read health insurance account and benefit information		Request and/or receive and read the information on health insurance benefits	
**Advanced functions**						
	**Medications management**					
		PH.3.4	Manage medications		Help patients manage his or her medications	
	**Communication (patient-provider communication)**					
		PH.6.3	Communications between provider and/or the PHR account holder’s representative		Capture information in preparation for a consultation and maintain continuous communications with the health provider	
		IN.3.10	Secure messaging		Enable secure electronic communication with health providers	
	**Appointment management**					
		PH.6.3	Communications between provider and/or the PHR account holder’s representative		Capture information in preparation for a consultation and maintain continuous communications with the health provider	
	**Education**					
		PH.4	Manage health education		Provide proper medical education and patient-specific knowledge based on information in the PHR	
	**Self-health monitoring**					
		PH.3.1	Manage personal clinical measurements and observations		Provide the patient capability to enter personally sourced data and make it available to authorized health providers or other users or applications	
**Supporting functions**						
	**Sharing**					
		IN.2	Standards-based interoperability		Interoperability standards enable the sharing of information between PHRs and other systems	
	**Access control**					
		IN.3.3	Entity access control		PHR must perform authentication and authorization of users or applications	
	**Audit logs and** **backup option**					
		IN.4	Auditable records		Provide system access and use audit capabilities to indicate who accessed the record, how, and when the action was taken	
	**Customization option**					
		IN.1.3	Present ad hoc views of the health record		Provide ad hoc views of the PHR information	
	**User guide**					
		PH.1.1	Identify and maintain a PHR account holder record		Offer user guide for the installation, initialization, registration, or operation of their PHR	

^a^HL7 PHR-S FM: Health Level 7 Personal Health Record System Functional Model.

^b^PHR: personal health record.

The functionality identified in this review covers the main section (PH, S, IN) in the HL7 PHR-S FM. However, functionalities and data elements found in this review are on the individual level that focuses on improving health care. Functions that are not included in this review are functions related to the secondary use of health data. Secondary health data use applies to personal health information for uses outside direct health care delivery [132]. In the HL7 PHR-S FM, a population health and wellness (PH 3.6) function helps control public health risks to the population and patients. For example, it enables patients to export anonymized data for biosurveillance and public health reporting, and patients can get alerts or warnings regarding population health threats. A manage other resources (S.4) function supports patient enrollment in clinical trials or research [131]. From this review, only a few studies [34,91] mentioned that PHRs could be used for secondary health data use, but they did not explain specific data needed for this function. A discussion about secondary health data use in PHRs can be an opportunity for future research.

Not all functions in the HL7 PHR-S FM were found in this review study because the HL7 PHR-S FM is universal and generic by design. There may be additional constraints in certain realms or regions. PHR developers or designers can create a functional profile to define a selected set of applicable functions for a particular purpose, group of users, degree of interoperability, or custodian [130]. This study defines PHR functionalities based on the current state of research and provides more examples of data elements and subfunctions for each functionality. This study also found that the HL7 PHR-S FM only includes patient-provider communication. Other communications, such as communication with others in a similar situation and support groups, are not discussed in the HL7 PHR-S FM.

### Limitations

This study is limited to reviewing the implementation of PHRs in research articles and does not address the implementation of commercial PHRs available on the internet. Thus, the functionalities and issues of the PHRs defined in this study may not reflect the state of the practice. This paper does not discuss which functions are more common or whether certain functions are used more frequently than others and does not discuss each function’s benefits and impact on health outcomes. We cannot determine which functionality should be prioritized in the implementation of PHR. We only discuss the functions that are generally mentioned in the selected paper. Each function’s data element may not be comprehensive and might not be generalizable to all patient populations. This is because each disease or condition has different specific data.

### Conclusions

This systematic literature review paper discussed functionalities and issues in the implementation of PHRs. Seven function categories are identified in this review, which are grouped into basic and advanced functions. In addition to these functionalities, other supporting functionalities may also need to be developed based on the issues identified in this study. Based on the results, this paper provides an integrated PHR architectural model that describes the functional requirements and data sources of PHRs. This study can offer recommendations or guidance in implementing PHRs by health care facilities management, application developers, policymakers, or other related stakeholders. Functionalities (including data elements and subfunctions) listed in this study and architectural model (Figure 10) can be used when considering what features to implement in a PHR. The model (Figure 10) can also serve as the target data sources to be integrated into the PHR system. Moreover, technological issues explained in this study can be used to develop policies in the implementation of PHRs. For example, since security and privacy are identified as technological issues in this study, implementers of PHRs should develop policies that govern access control in PHRs. The findings of this study may be translated as functional and nonfunctional requirements of the PHR system. This study’s findings can also serve as a basis and comparison for other researchers who will examine PHR functionality and use in the future. PHR integrated architecture (Figure 10) can be used as a model that other researchers can use to compare, map, or evaluate the PHR functionalities that will be examined. Furthermore, personal factors such as age, culture, and health and technology literacy levels can influence security, privacy, and usability issues. Future studies can be conducted to analyze the effect of personal factors on technological issues.

## Appendix

Multimedia Appendix 1 PRISMA checklist.

Multimedia Appendix 2 Characteristics of the included studies.

Multimedia Appendix 3 Data elements and subfunctions.

## Abbreviations

EHR: electronic health record
FHIR: Fast Healthcare Interoperability Resources
HL7: Health Level 7
HL7 PHR-S FM: Health Level 7 Personal Health Record System Functional Model
IN: information infrastructure
PH: personal health
PHR: personal health record
PRISMA: Preferred Reporting Items for Systematic reviews and Meta-analyses
S: supportive
