# Supplementary material for: Functionalities and Issues in the Implementation of Personal Health Records: Systematic Review
Source: J Med Internet Res. 2021 Jul 21;23(7):e26236. doi: 10.2196/26236 (PMC8339989; doi:10.2196/26236)
Supplement: Multimedia Appendix 2 [file jmir_v23i7e26236_app2.docx]

**Multimedia Appendix 2.** Characteristics of the included studies.

| **No** | **Author(s) and Year** | **Title** | **Sources** | **Methodology** | **Type of Study** | **Participants** | **Study Location** | **Type of PHR** | **Purpose of PHR** |
| --- | --- | --- | --- | --- | --- | --- | --- | --- | --- |
|  | Abd-alrazaq et al. (2019) | Factors affecting patients’ use of electronic personal health records in England: cross-sectional study | Journal of Medical Internet Research | Quantitative | Questionnaire | Patients | UK | Tethered | General |
|  | Abdekhoda et al. (2019) | The effect of confidentiality and privacy concerns on adoption of personal health record from patient’s perspective | Health and Technology | Quantitative | Questionnaire | Patients | Iran | Stand-alone | General |
|  | Abdulnabi et al. (2017) | A distributed framework for health information exchange using smartphone technologies | Journal of Biomedical Informatics | Conceptual paper | Conceptual paper | No participants | Malaysia | Tethered | General |
|  | Aliakbarpoor et al. (2017) | Designing a HL7 compatible personal health record for mobile devices | International Forum on Research and Technologies for Society and Industry (RTSI) | Conceptual paper | Conceptual paper | No participants | Italy | Tethered | Cardiovascular disease |
|  | Alpert et al. (2016) | Applying Multiple Methods to Comprehensively Evaluate a Patient Portal’s Effectiveness to Convey Information to Patients | Journal of Medical Internet Research | Qualitative | Interview and focus group | Patients and Clinicians | US | Tethered | General |
|  | Arcia (2017) | Time to push: use of gestational age in the electronic health record to support delivery of relevant prenatal education content | eGEMs (Generating Evidence & Methods to improve patient outcomes) | Qualitative | Focus group | Patients | US | Tethered | Pregnancy |
|  | Arcury et al. (2017) | Patient portal utilization among ethnically diverse low income older adults: observational study | JMIR Medical Informatics | Quantitative | Questionnaire | Patients | US | Tethered | Older adults |
|  | Avdagovska et al. (2020) | Patient Portal Implementation and Uptake: Qualitative Comparative Case Study | Journal of Medical Internet Research | Qualitative | Interview | Patients and healthcare providers | Canada | Tethered | General |
|  | Barbarito et al. (2015) | Implementing the lifelong personal health record in a regionalised health information system: the case of Lombardy, Italy | Computers in Biology and Medicine | Conceptual paper | Conceptual paper | No participants | Italy | Integrated | General |
|  | Baudendistel et al. (2015) | Personal electronic health records: Understanding user requirements and needs in chronic cancer care | European journal of cancer care | Qualitative | Focus Group | Physicians and patients | German | Tethered | Chronic disease |
|  | Baudendistel et al. (2016) | Cross-sectoral cancer care: views from patients and health care professionals regarding a personal electronic health record | European journal of cancer care | Qualitative | Focus group | Physicians and patients | German | Tethered | Colorectal Cancer |
|  | Bernhard et al. (2018) | Developing a Shared Patient-Centered, Web-Based Medication Platform for Type 2 Diabetes Patients and Their Health Care Providers: Qualitative Study on User Requirements. | Journal of Medical Internet Research | Qualitative | Focus group | Patients and healthcare providers | German | Tethered | Chronic disease |
|  | Brandner et al. (2016) | The patient portal of the personal cross- enterprise electronic health record (PEHR) in the Rhine-Neckar-Region | Studies in Health Technology and Informatics | Conceptual Paper | Conceptual Paper | No participants | German | Integrated | General |
|  | Brown et al. (2016) | Preferences of current and potential patients and family members regarding implementation of electronic communication portals in intensive care units | Annals of the American Thoracic Society | Quantitative | Questionnaire | Patients | US | Tethered | ICU patients |
|  | Cabitza et al. (2015) | User-driven prioritization of features for a prospective InterPersonal Health Record: perceptions from the Italian context | Computers in Biology And Medicine | Mixed-method | Interview and Questionnaire | Physicians and patients | Italy | Tethered | General |
|  | Carryer et al. (2017) | Patients’ experience and understanding of E-portals in rural general practice: an ethnographic exploration | Journal of Primary Health Care | Qualitative | Interview | Patients | New Zealand | Integrated | General |
|  | Cernian et al (2020) | PatientDataChain: A Blockchain-Based Approach to Integrate Personal Health Records. | Sensors | Conceptual Paper | Conceptual Paper | No participants | Romania | Integrated | General |
|  | Clarke et al. (2020) | Usability and cognitive load in the design of a personal health record | Health Policy and Technology | Mixed-method | Interview and questionnaire | Patients | US | Stand-alone | Cardiovascular disease |
|  | Collins et al (2017) | Acute care patient portals: a qualitative study of stakeholder perspectives on current practices. | Journal of the American Medical Informatics Association | Mixed-method | surveys, interviews, focus groups, and site visits | Patients and Families, Clinician Leaders, Policymakers and Administrators Information Systems Leaders, Researchers | US | Tethered | Hospital patients |
|  | Conway et al. (2019) | User experiences of an electronic personal health record for diabetes | Journal of Diabetes Science and Technology | Quantitative | Questionnaire | Patients | UK | Tethered | Diabetes |
|  | Cronin et al (2018) | Patient and healthcare provider views on a patient-reported outcomes portal | Journal of the American Medical Informatics Association | Quantitative | Questionnaire | Patients | US | Tethered | General |
|  | Davis and MacKay (2020) | Moving beyond the rhetoric of shared decision-making: designing personal health record technology with young adults with type 1 diabetes | Canadian Journal of Diabetes | Qualitative | User-centered design using Interview and Task to Function Mapping | Patients, healthcare providers | Canada | Integrated | Type I diabetes |
|  | Dexheimer et al. (2019) | Sharing personal health record data elements in protective custody: youth and stakeholder perspectives | Journal of the American Medical Informatics Association | Mixed-method | Participatory research design using concept mapping | Caseworkers, community members, foster youth, and healthcare providers | US | Tethered | Foster youth |
|  | Eschler et al (2016) | Integrating the patient portal into the health management work ecosystem: user acceptance of a novel prototype. | Annual Symposium proceedings. AMIA Symposium | Qualitative | Interview | Patients and caregivers | US | Tethered | Chronic disease |
|  | Farinago et al. (2018) | Human-centered design of a personal health record system for metabolic syndrome management based on the ISO 9241-210:2010 standard | Journal of Multidisciplinary Healthcare | Mixed-method | Questionnaire, interviews, and focus group | Patients | Colombia | Stand-alone | Metabolic syndrome |
|  | Fernando et al (2019) | An Overall Health and Well-Being Data Model for Employer-Sponsored Personal Health Records | ACM International Conference Proceeding Series | Mixed-method | Focus Group and Questionnaire | Patients | Sri Lanka | Tethered | Other population |
|  | Fernando et al. (2020) | Design guidelines for effective occupation-based personal health records | Proceedings of the Australasian Computer Science Week Multiconference (ACSW 2020) | Mixed-method | Focus group and questionnaire | Patients | Sri Lanka | Tethered | Employee |
|  | Fonseca et al. (2019) | Smart mobile computing in pregnancy care | Proceedings of 34th International Conference on Computers and Their Applications (CATA 2019) | Conceptual Paper | Conceptual Paper | No participants | Portuguese | Stand-alone | Pregnancy |
|  | Forchuk et al. (2015) | Client perceptions of the mental health engagement network: a qualitative analysis of an electronic personal health record | BMC Psychiatry | Qualitative | Focus group | Patients | Canada | N/A | Mental health |
|  | Fosser et al. (2019) | User -centered design of a patient medication reconciliation module in an integrated personal health record | Studies in Health Technology and Informatics | Qualitative | Interview | Physicians and patients | Argentina | Tethered | Outpatient |
|  | Fuji et al (2015) | A Qualitative Study of How Patients with Type 2 Diabetes Use an Electronic Stand-Alone Personal Health Record | Telemedicine and e-Health | Qualitative | Interview | Patients | US | Stand-alone | Chronic disease |
|  | Fylan et al. (2018) | Making it work for me: beliefs about making a personal health record relevant and useable | BMC Health Services Research | Qualitative | Focus Group | Patients | UK | N/A | General |
|  | Gagnon et al. (2016) | Adoption of electronic personal health records in Canada: perceptions of stakeholders | International Journal of Health Policy and Management | Qualitative | Interview | Patients, administrators, health professionals, health technology organizations, government, researchers | Canada | N/A | General |
|  | Gee et al (2015) | e-Patients perceptions of using personal health records for self-management support of chronic illness | CIN - Computers Informatics Nursing | Qualitative | Interview | Patients | US | Tethered | Chronic disease |
|  | Geerts et al (2019) | Developing a patient portal for haematology patients requires involvement of all stakeholders and a customised design, tailored to the individual needs. | BMC medical informatics and decision making | Mixed-method | Focus Group and Questionnaire | Patients and healthcare providers | Netherlands | Tethered | Chronic disease |
|  | Griffin et al. (2016) | Patient portals: who uses them? what features do they use? and do they reduce hospital readmissions? | Applied Clinical Informatics | Quantitative | Questionnaire | Patients | US | Tethered | Chronic diseases |
|  | Gupta et al. (2015) | mSwasthya: a mobile-enabled personal health record management system | International Conference on Computing, Communication and Automation (ICCCA2015) | Conceptual Paper | Conceptual Paper | Patients | India | Integrated | General |
|  | Hanna et al (2017) | Patient perspectives on a personally controlled electronic health record used in regional Australia: ‘I can be like my own doctor’ | Health Information Management Journal | Qualitative | Interview | Patients | Australia | Integrated | General |
|  | Heuvel et al. (2018) | The user experiences and clinical outcomes of an online personal health record to support self-management of bipolar disorder: a pretest-posttest pilot study | Journal of Affective Disorders | Quantitative | Questionnaire | Patients, clinicians | Netherlands | Stand-alone | Bipolar disorder |
|  | Hill et al. (2018) | Potential of personal health record portals in the care of individuals with spinal cord injuries and disorders: provider perspectives | The Journal of Spinal Cord Medicine | Qualitative | Interview | Healthcare providers | US | Tethered | Spinal cord injuries and disorders (SCI/D) |
|  | Iacona et al. (2015) | Personal health system: A tool to support the patient empowerment | E-Health and Bioengineering Conference (EHB) | Conceptual Paper | Conceptual Paper | No participants | Italy | Integrated | General |
|  | Iatraki et al. (2018) | Personal health information recommender: implementing a tool for the empowerment of cancer patients. | Ecancermedicalscience | Conceptual Paper | Conceptual Paper | Physicians and patients | European Union | Integrated | Cancer |
|  | Javier et al. (2019) | Racial and ethnic disparities in use of a personal health record by veterans living with HIV | Journal of the American Medical Informatics Association | Quantitative | Cohort study | Patients | US | Tethered | HIV |
|  | Jing (2016) | An empirical study on the features influencing users’ adoption towards personal health records system | International Conference on Service Systems and Service Management (ICSSSM) | Quantitative | Questionnaire | Patients | China | N/A | General |
|  | Jones et al. (2015) | The wired patient: patterns of electronic patient portal use among patients with cardiac disease or diabetes | Journal of Medical Internet Research | Quantitative | Cohort study | Patients | US | Tethered | Chronic illnesses |
|  | Jung et al (2017) | Support for sustainable use of personal health records: Understanding the needs of users as a first step towards patient-driven mobile health | JMIR mHealth and uHealth | Quantitative | Questionnaire | Patients | South Korea | Tethered | General |
|  | Katehakis et al (2017) | Integrated care solutions for the citizen: personal health record functional models to support interoperability | European Journal for Biomedical Informatics | Conceptual Paper | Conceptual Paper | No participants | European Union | Integrated | General |
|  | Khaneghah et al. (2016) | Users’ attitudes towards personal health records: a cross-sectional pilot study | Applied Clinical Informatics | Mixed-method | Interview and questionnaire | Patients | Canada | Tethered | Type II diabetes |
|  | Kipping et al. (2016) | A web-based patient portal for mental health care: benefits evaluation | Journal of Medical Internet Research | Quantitative | Questionnaire | Patients | Canada | Tethered | Mental health |
|  | Lafata et al (2018) | Patients' Adoption of and Feature Access Within Electronic Patient Portals | American Journal of Managed Care | Quantitative | Cohort study | Patients | US | Tethered | General |
|  | Lalitaphanit and  Theeraroungchaisri (2016) | Factors affecting community pharmacy customers’ decision to use personal health records via smartphone | Thai Journal of Pharmaceutical Sciences (TJPS) | Quantitative | Questionnaire | community pharmacy customers | Thailand | Stand-alone | General |
|  | Latulipe et al (2015) | Design Considerations for Patient Portal Adoption by Low-Income, Older Adults. | Proceedings of the SIGCHI conference on human factors in computing systems. | Qualitative | Interview | Patients and healthcare providers | US | Tethered | Older adults |
|  | Marchak et al. (2019) | User-centered design and enhancement of an electronic personal health record to support survivors of pediatric cancers | Supportive Care in Cancer | Qualitative | Focus Group, Interview | Patient, health provider | US | Stand-alone | Pediatric cancers |
|  | Marthick et al (2019) | Feasibility of an Interactive Patient Portal for Monitoring Physical Activity, Remote Symptom Reporting, and Patient Education in Oncology: Qualitative Study. | JMIR cancer | Qualitative | Interview | Patients | Australia | Integrated | Chronic disease |
|  | McAlearney et al (2019) | Patients' perceptions of portal use across care settings: Qualitative study | Journal of Medical Internet Research | Qualitative | Interview | Patients | US | Tethered | Hospital patients |
|  | Metting et al (2018) | Assessing the Needs and Perspectives of Patients With Asthma and Chronic Obstructive Pulmonary Disease on Patient Web Portals: Focus Group Study | JMIR Formative Research | Qualitative | focus group | Patients | Netherlands | Tethered | Chronic disease |
|  | Minoletti et al. (2019) | User-centered design of a pediatric vaccination module for patients | Studies in Health Technology and Informatics | Mixed-method | Interview and Questionnaire | Patients | Argentina | Tethered | Pediatric vaccination |
|  | Mishra et al. (2019) | Qualitative and quantitative analysis of patients’ perceptions of the patient portal experience with OpenNotes | Applied Clinical Informatics | Quantitative | Questionnaire | Patients | US | N/A | General |
|  | Nazi et al (2018) | A Decade of Veteran Voices: Examining Patient Portal Enhancements Through the Lens of User-Centered Design | Journal of Medical Internet Research | Quantitative | Questionnaire | Patients | US | Tethered | General |
|  | O’Leary et al. (2016) | Patients’ and healthcare providers’ perceptions of a mobile portal application for hospitalized patients | BMC Medical Informatics and Decision Making | Qualitative | Focus Group, Interview | Patient, health provider | US | Tethered | Hospitalized patients |
|  | Pang et al (2020) | Privacy concerns of the Australian My Health Record: Implications for other large-scale opt-out personal health records | Information Processing and Management | Mixed-method | computational analysis and Qualitative | Academic, Clinician, IT, Law, Media, government, patients | Australia | Integrated | General |
|  | Park et al. (2018) | Managing Patient-Generated Health Data Through Mobile Personal Health Records: Analysis of Usage Data | JMIR mHealth and uHealth | Quantitative | Cohort study | Patients | South Korea | Stand-alone | General |
|  | Ploner et al. (2019) | Concept to gain trust for a German personal health record system using public cloud and FHIR | Journal of Biomedical Informatics | Mixed-method | Interview and Questionnaire | Physicians and patients | German | Integrated | General |
|  | Pohlmann et al. (2020) | Digitalizing health services by implementing a personal electronic health record in Germany: qualitative analysis of fundamental prerequisites from the perspective of selected experts | Journal of Medical Internet Research | Qualitative | Interview | Healthcare professionals, eHealth experts | German | Tethered | General |
|  | Portz et al. (2019) | Using the technology acceptance model to explore user experience, intent to use, and use behavior of a patient portal among older adults with multiple chronic conditions: descriptive qualitative study | Journal of Medical Internet Research | Qualitative | Focus Group | Patients | US | Tethered | Multiple chronic conditions (MCC) |
|  | Poss-Doering et al. (2018) | Utilizing a prototype patient-controlled electronic health record in Germany: qualitative analysis of user-reported perceptions and perspectives | Journal of Medical Internet Research | Qualitative | Interview | Physicians and patients | German | Tethered | Gastrointestinal cancer |
|  | Rau et al. (2017) | Importance-performance analysis of personal health records in Taiwan: A web-based survey | Journal of Medical Internet Research | Quantitative | Questionnaire | Patients | Taiwan | Tethered | General |
|  | Redelmeier and Kraus (2018) | Patterns in patient access and utilization of online medical records: analysis of MyChart | Journal of Medical Internet Research | Quantitative | Cohort study | Patients | Canada | Tethered | General |
|  | Rezaee et al. (2018) | Designing and implementation of web-based personal health record for patients with inflammatory bowel disease | Govaresh | Quantitative | Questionnaire | Patients | Iran | Tethered | Inflammatory bowel disease (IBD) |
|  | Rief et al. (2017) | Using health information technology to foster engagement: patients’ experiences with an active patient health record | Health Communication | Qualitative | Focus group | Patients | US | N/A | Cardiovascular disease |
|  | Robinson et al (2016) | Use of a patient portal during hospital admissions to surgical services | AMIA ... Annual Symposium proceedings. AMIA Symposium | Quantitative | Cohort study | Patients | US | Tethered | Hospital patients |
|  | Roehrs et al. (2017) | OmniPHR: A distributed architecture model to integrate personal health records | Journal of Biomedical Informatics | Conceptual Paper | Conceptual Paper | No participants | Brazil | Integrated | General |
|  | Ruhi and Majedi (2015) | Consumer adoption of personal health records: an empirical investigation of personal & technology factors | International Conferences e-Health 2015, e-Commerce and Digital Marketing 2015 and Information Systems Post-Implementation and Change Management 2015 | Quantitative | Questionnaire | Patients | US | N/A | General |
|  | Ryan et al (2016) | Implementing and Using a Patient Portal: A qualitative exploration of patient and provider perspectives on engaging patients. | Journal of innovation in health informatics | Qualitative | Interview | patients and health care professionals | Canada | Tethered | Chronic disease |
|  | Ryu et al. (2017) | Impact of an Electronic Health Record-Integrated Personal Health Record on Patient Participation in Health Care: Development and Randomized Controlled Trial of MyHealthKeeper | Journal of Medical Internet Research | Quantitative | Randomized clinical trial (RCT) | Patients | South Korea | Tethered | General |
|  | Samet et al. (2018) | Privacy-preserving personal health record (P3HR): A secure android application | ACM International Conference Proceeding Series | Conceptual Paper | Conceptual Paper | - | Canada | Tethered | General |
|  | Saripalle et al. (2019) | Using HL7 FHIR to achieve interoperability in patient health record | Journal of Biomedical Informatics | Conceptual Paper | Conceptual Paper | - | US | Tethered | General |
|  | Schladen et al. (2017) | Exploration of the Personal Health Record as a Tool for Spinal Cord Injury Health Self-Management and Coordination of Care | Topics in Spinal Cord injury Rehabilitation | Qualitative | Interview | Patients | US | Integrated | Spinal cord injuries |
|  | Sheehan & Lucero (2015) | Initial Usability and Feasibility Evaluation of a Personal Health Record-Based Self-Management System for Older Adults | eGEMs (Generating Evidence & Methods to Improve Patient outcomes) | Mixed-method |  | Patients | US | Tethered | Older Adults |
|  | Sieverink et al. (2019) | Evaluating the implementation of a personal health record for chronic primary and secondary care: a mixed methods approach | BMC Medical Informatics and Decision Making | Mixed-method | Log data, focus group, interviews | Patients, caregivers | Netherlands | N/A | Chronic ilnesses |
|  | Smith et al. (2017) | Lessons learned after redesigning a personal health record | Studies in Health Technology and Informatics | Qualitative | Focus group | Patients | Argentina | Tethered | General |
|  | Song et al (2017) | Standard-based patient-centered personal health record system | Proceedings of the 11th International Conference on Ubiquitous Information Management and Communication (IMCOM 2017) | Conceptual Paper | Conceptual Paper | No participants | US | Integrated | General |
|  | Strudwick et al (2020) | Identifying indicators of meaningful patient portal use by psychiatric populations. | Informatics for health & social care | Qualitative | Focus group | Patients | Canada | Tethered | Mental Health |
|  | Subbe et al. (2019) | Digital technology: opportunities and barriers for usage of personal health records in hospital – report from a ­workshop of the health informatics unit at the royal college of physicians | Future Healthcare Journal | Qualitative | Focus group | Physicians and patients | UK | N/A | Women |
|  | Tao et al (2018) | Factors Affecting Consumer Acceptance of an Online Health Information Portal among Young Internet Users | CIN - Computers Informatics Nursing | Quantitative | Questionnaire | Patients | China | Tethered | General |
|  | Tarver et al. (2019) | Usefulness and usability of a personal health record and survivorship care plan for colorectal cancer survivors: survey study | Journal of Medical Internet Research | Quantitative | Questionnaire | Patients | US | Stand-alone | Colorectal Cancer |
|  | Tieu et al. (2015) | Barriers and facilitators to online portal use among patients and caregivers in a safety net health care system: a qualitative study | Journal of Medical Internet Research | Qualitative | Interview | Patients and Caregivers | US | Tethered | Chronic illnesses |
|  | Tulu et al. (2016) | An analysis of patient portal utilization: what can we learn about online patient behavior by examining portal click data? | Health Systems | Quantitative | Cohort study | Patients | US | Tethered | General |
|  | Turner et al (2015) | Use of Patient Portals for Personal Health Information Management: The Older Adult Perspective | AMIA Annual Symposium Proceedings | Qualitative | Interview | Patients | US | Tethered | Older adults |
|  | Urbauer et al. (2015) | Applicability of IHE/Continua components for PHR systems: learning from experiences | Computers in Biology and Medicine | Qualitative | Interview | General population, healthcare providers, software vendors, hospital IT officers, health regulators, and  PHR/EHR experts | Austria | Integrated | General |
|  | Van den Bulck et al (2018) | Designing a Patient Portal for Patient-Centered Care: Cross-Sectional Survey | Journal of Medical Internet Research | Quantitative | Questionnaire | Patients | Belgium | N/A | General |
|  | Vassilakopoulou et al. (2018) | Between personal and common: the design of hybrid information spaces | Computer Supported Cooperative Work (CSCW) | Qualitative | Interview | Project team | Norway | Tethered, Standalone | General |
|  | Vimalachandran et al (2020) | Improving accessibility of the Australian My Health Records while preserving privacy and security of the system | Health Information Science and Systems | Conceptual Paper | Conceptual Paper | No participants | Australia | Integrated | General |
|  | Voigt et al (2020) | A Digital Patient Portal for Patients With Multiple Sclerosis. | Frontiers in Neurology | Mixed-method | Questionnaire and workshops | patients and health care professionals | German | Tethered | Chronic disease |
|  | Walker et al (2018) | Optimizing the User Experience: Identifying Opportunities to Improve Use of an Inpatient Portal | Applied clinical informatics | Qualitative | Interview | Patients | US | Tethered | Hospital patients |
|  | Walsh (2017) | The e-health literacy demands of Australia’s my health record: a heuristic evaluation of usability | Perspectives in Health Information Management | Quantitative | Questionnaire | Researchers | Australia | N/A | General |
|  | Wang and Dolozel (2016) | Usability of web-based personal health records: an analysis of consumers’ perspectives | Perspectives in Health Information Management | Quantitative | Questionnaire | Patients | US | Stand-alone | General |
|  | Wang et al. (2018) | Design and implementation of personal health record systems based on knowledge graph | International Conference on Information Technology in Medicine and Education (ITME) | Conceptual Paper | Conceptual Paper | - | China | Tethered | General |
|  | Wells et al. (2019) | Perspectives of New Zealand patients and GPs at the beginning of patient portal implementation | Journal of Primary Health Care | Quantitative | Questionnaire | Patients, GPs | New Zealand | Tethered | General |
|  | Wilcox et al. (2016) | Interactive tools for inpatient medication tracking: a multi-phase study with cardiothoracic surgery patients | Journal of the American Medical Informatics Association | Qualitative | Interview | Patients, Pharmacists | US | Tethered | Inpatient (cardiothoracic surgery patients) |
|  | Wildenbos et al (2018) | Older adults using a patient portal: registration and experiences, one year after implementation | Digital health | Quantitative | Questionnaire | Patients | US | Tethered | Older adults |
|  | Woollen et al (2016) | Patient Experiences Using an Inpatient Personal Health Record | Applied clinical informatics | Qualitative | Interview | Patients | US | Tethered | Hospital patients |
|  | Wright et al. (2016) | Missing links: challenges in engaging the underserved with health information and communication technology | Proceedings of the 10th EAI International Conference on Pervasive Computing Technologies for Healthcare | Qualitative | Interview | Patients, providers, administrators, and staff | US (Indiana) | Tethered | General |
|  | Yen et al. (2018) | Usability evaluation of a commercial inpatient portal | International Journal of Medical Informatics | Qualitative | Interview | Patients | US | N/A | Inpatient |
|  | Zhou et al. (2019) | Applying a User-Centered Approach to Building a Mobile Personal Health Record App: Development and Usability Study | Journal of Medical Internet Research | Quantitative | Questionnaire | Patients | US | Stand-alone | General |
