# Supplementary material for: Functionalities and Issues in the Implementation of Personal Health Records: Systematic Review
Source: J Med Internet Res. 2021 Jul 21;23(7):e26236. doi: 10.2196/26236 (PMC8339989; doi:10.2196/26236)
Supplement: Multimedia Appendix 3 [file jmir_v23i7e26236_app3.docx]

**Multimedia Appendix 3**. Data elements and subfunctions.

| **Function** | **Sub-Function** | **Data Element** | **Reference** |  |
| --- | --- | --- | --- | --- |
| **Basic Function** | | | | |
| Health Record | Current health record | problem lists | [22,24–29,45,56,67–71] |  |
|  |  | allergies | [22,25–28,30–33,35–39,56,67–70] |  |
|  |  | immunization | [22,26–28,30,32–35,40–42,56,68–71] |  |
|  |  | laboratory and test results | [22,24,26–30,33,36,37,40,42–61,67–71] |  |
|  |  | diagnostic information | [32,37,44,45,62] |  |
|  |  | discharge information | [31,34,63] |  |
|  |  | clinical notes | [24,30,33,42,50,61,64,67,69] |  |
|  | Past health record | medical history | [22,24,30,33,35,37,39,45,51,57,64,65,67,68] |  |
|  |  | family history | [28,30,32,33,35,37,66] |  |
|  |  | genetic history | [45] |  |
|  |  | surgical history | [26,28,33,35,45,66,68] |  |
|  |  | social history | [32,33,35,37,45,68] |  |
| Administrative Record | Patient profile | name | [25,39,44,72,73] |  |
|  |  | gender | [25,26,44,72] |  |
|  |  | birthdate | [25,26,44,72,73] |  |
|  |  | blood type | [39,44] |  |
|  |  | contact information | [25,31,68,72] |  |
|  |  | parent’s names | [25,45] |  |
|  |  | change password, address, and email address | [22] |  |
|  | Health professionals profile | health workers’ name | [34,39,55,74] |  |
|  |  | role | [74,75] |  |
|  |  | educational background | [75] |  |
|  |  | contact information | [42,45] |  |
|  |  | specialty | [76] |  |
|  |  | location | [76] |  |
|  |  | pictures | [46,55,59,74,77,78] |  |
|  | Hospital information | location, contact info, address, navigation | [70] |  |
|  | View and pay bill | | [42,55,77] |  |
|  | Insurance-related information | | [22,30,34,45,66] |  |
| **Advanced Function** | | | | |
| Medications Management | Current and past medications | list of current medications | [25,28,29,35,38,45,46,52,56,59,60,68,69] |  |
|  |  | medication name and dosage | [32,35,40,60,74,77,79,80] |  |
|  |  | list of past medications | [28,29,36,42,45,46,60,69,70,73,81] |  |
|  |  | purpose or class of medications | [74] [80] |  |
|  | Prescriptions management | list of prescribed medications | [26,30,39,54,79,82,83] |  |
|  |  | prescribing physician | [79] |  |
|  |  | refill prescription | [24,27,29–31,33,34,36,39,48,53,55,57,60,61,84] |  |
|  |  | order medications | [29,39,71] |  |
|  |  | deliver purchased medication | [79] |  |
|  |  | track the delivery of medication | [36,39,42] |  |
|  | Medication schedulers and reminders | | [28,70,83,85] |  |
|  | Drug or medicine reconciliation | | [42,51,63,83] |  |
|  | Warning alerts of potential adverse interactions | | [38,68,73] |  |
| Communication | Patient-provider communication | messaging | [23,27,29–32,36,38,40,42,43,46,48,49,51–53,55–57,59,61,67,69,71,74,75,82–84,86–92] |  |
|  |  | comments or questions text box | [74,80] |  |
|  | Other communication | communication with others in a similar situation | [28,29,83,88] |  |
|  |  | communication with support group | [62,87] |  |
|  |  | communication with family | [75,89] |  |
|  |  | communication with customer support and billing departments | [22] |  |
|  | Record of past conversations | | [36] |  |
|  | Email or text notification | | [24,93,94] |  |
|  | Tracking the status of a question | | [80] |  |
|  | Message multiple providers | | [24] |  |
|  | Import selected emails and interactions on the social network | | [86] |  |
| Appointment Management | Request or schedule appointments | | [22,23,25–28,30,33,34,40,42,48–50,52,55,57,60,61,67,71,85,87,92] |  |
|  | Past and upcoming appointments | | [29,31,36,51,53,56,59,63,64,73,77] |  |
|  | Reminders or notifications for upcoming appointments | | [33,42,47,48,60,61,81,94] |  |
|  | Calendar | | [34,78] |  |
| Education | Information sources | trusted website | [45,90] |  |
|  |  | health information libraries | [22,30] |  |
|  |  | video resources | [46,59,95] |  |
|  |  | government supported information | [95] |  |
|  | Type of information | lifestyle management | [45,57,71] |  |
|  |  | first-aid information | [40,70] |  |
|  |  | discharge instructions | [31] |  |
|  |  | physical activities guidance | [77] |  |
|  | Health-specific information | pregnancy | [97,98] |  |
|  |  | mental health | [45,61] |  |
|  |  | chronic diseases-related education | [90,95] |  |
|  | Intelligent search engine | | [99] |  |
| Self-Health Monitoring | Nutrition and diet information | weight | [30,33,39,58,66,67,72,85,86,90,98,100–102] |  |
|  |  | height | [39,58,66] |  |
|  |  | physical activity or exercise | [30,33,58,66,70,96,98,100,101] |  |
|  |  | food and meals | [33,66,98] |  |
|  | Vital sign | temperature | [26,44] |  |
|  |  | blood pressure | [30,33,44,58,66,67,70,72,85,90,98,101,103] |  |
|  |  | blood glucose | [30,58,66,70,72,85,86,98,103] |  |
|  |  | heart rate | [90] |  |
|  | Other | sleep | [33,66,95,100,101] |  |
|  |  | period | [33,100] |  |
|  |  | moods | [98,100,101] |  |
|  |  | stress | [66,70,100] |  |
|  | Health calculation | body mass index | [39,66,70,85,100] |  |
|  |  | body fat percentage | [70] |  |
|  |  | waist to height ratio | [70] |  |
|  |  | calorie | [70] |  |
|  |  | cholesterol level | [66,86] |  |
|  |  | glycemia | [86] |  |
|  | Disease risks calculation | | [85] |  |
|  | Data visualization | | [30,33,70,72,81,86,101,102,105] |  |
